# Orthosomnia: When Perfect Sleep Becomes an Obsession

**DOI:** 10.7759/cureus.119332

**Published:** 2026-11-08

**Authors:** Binh Pham

**Affiliations:** 1 Psychiatry, Western University of Health Sciences, Pomona, USA; 2 Sleep Medicine, Amen Clinics, Costa Mesa, USA; 3 Sleep Medicine, Southwest Healthcare Medical Education Consortium, Temecula, USA

**Keywords:** consumer sleep technology, insomnia, orthosomnia, sleep anxiety, sleep effort, sleep health, sleep optimization, sleep perfectionism, sleep tracking, wearable technology

## Abstract

Orthosomnia describes an excessive preoccupation with achieving ideal sleep, often reinforced by consumer sleep technologies and device-generated metrics. As sleep becomes increasingly measured, scored, and optimized, information intended to improve sleep may instead contribute to vigilance, rigidity, and sleep-related stress. This creates a paradox consistent with the attention-intention-effort model of insomnia: sleep depends partly on automaticity and reduced arousal, yet the pursuit of perfect sleep may increase monitoring, effort, and concern about normal night-to-night variation. Consumer sleep technologies can provide useful information, but their outputs may acquire disproportionate authority when interpreted as definitive judgments about sleep quality. Orthosomnia may therefore extend beyond wearable use itself, reflecting a broader pattern of perfectionism, behavioral rigidity, and excessive sleep effort. Clinically, the important question may be whether sleep data remain informative or have begun to govern behavior. For selected patients, management may require less monitoring, greater behavioral flexibility, and renewed emphasis on daytime function and subjective restoration. Perfect sleep may become most elusive when normal variation is treated as failure.

## Editorial

Sleep is increasingly treated as something to measure, score, and optimize. Consumer sleep technologies can estimate sleep duration, awakenings, sleep stages, and other nightly metrics, transforming a largely automatic biological process into a performance target. For many individuals, these data may be useful. For others, however, greater access to sleep information may create new sources of concern rather than reassurance. Orthosomnia was originally described as an excessive preoccupation with obtaining ideal sleep, often accompanied by efforts to improve device-generated sleep metrics [1]. More recent work developing and validating the Bergen Orthosomnia Scale identified two dimensions, interference and rigidity, both of which were positively associated with sleep effort, perfectionism, obsessive-compulsive symptoms, and health anxiety [2]. Orthosomnia is not yet established as a distinct clinical disorder and may be better understood as a behavioral phenotype arising from these vulnerabilities, with excessive attention, intention, and effort providing a plausible mechanism for its persistence. It may therefore represent more than excessive interest in wearable data; it may reflect a loss of behavioral flexibility in the pursuit of sleeping correctly.

The paradox is that sleep cannot simply be perfected through greater effort. Normal sleep depends partly on automaticity and reduced vigilance, whereas excessive attention to sleep, deliberate attempts to control it, and repeated efforts to improve it may increase arousal. This is consistent with the attention-intention-effort model of insomnia, in which heightened monitoring, stronger intention to make sleep occur, and greater effort to achieve it can perpetuate sleep difficulty [3]. Technology may amplify this process by providing continuous feedback about a biological function that was once experienced largely without measurement. Each score, awakening, or deviation from an ideal target can become another signal requiring interpretation and correction. The hidden stressor may therefore be less the measurement itself than the perceived obligation to act on it. More information can reduce uncertainty to a point, but beyond that point it may become another form of surveillance.

The increasing sophistication of consumer sleep technology makes this paradox especially important. Validation studies suggest that many devices can provide useful estimates of sleep, but performance varies across devices and measures. Sleep detection is generally stronger than wake detection, while estimates of individual sleep stages remain less consistent [4]. These discrepancies may become particularly unsettling when device output conflicts with subjective experience. A person may feel rested yet receive a poor sleep score, or believe they slept continuously while the device reports repeated awakenings. Because users generally cannot see how proprietary algorithms convert physiologic signals into composite scores and stage estimates, they may have little ability to reconcile these outputs with how they actually feel. The problem, therefore, is not simply whether a device is accurate or inaccurate. Even reasonably useful estimates can acquire disproportionate authority when interpreted as definitive judgments about whether a night of sleep was good or bad. The data no longer merely describe sleep; they begin to shape how sleep is experienced.

This shift can gradually change the individual’s relationship with sleep. Subjective restoration and daytime functioning may become secondary to whether nightly metrics meet an expected standard. Repeated checking, increasingly rigid routines, extending time in bed, or changing behavior in response to minor fluctuations can reinforce the belief that every imperfect night requires correction. Each behavior may appear reasonable in isolation, yet together they can transform sleep from a passive biological process into a nightly performance task. The clinically relevant question may therefore be less whether a patient tracks sleep and more whether the data remain informative or have begun to govern behavior. When a score determines how a person feels about sleep more strongly than actual daytime functioning, optimization may have crossed into preoccupation.

The clinical response to orthosomnia may therefore require less optimization, not more. Current guidance on consumer sleep technology cautions against overreliance on device-generated information and emphasizes that these technologies do not replace clinical evaluation [5]. Practical strategies may include structured tech-free periods before bedtime, temporarily reducing or suspending review of nightly sleep scores, and discouraging compensatory extension of time in bed after an unfavorable reading. Stimulus control principles may help restore the association between bed and sleep rather than monitoring or effort, while paradoxical intention may be considered when excessive attempts to force sleep become prominent. For selected patients, restoring behavioral flexibility and redirecting attention toward daytime function and subjective well-being may be more useful than pursuing increasingly precise sleep targets. The broader lesson extends beyond wearables: technological progress gives us unprecedented access to human physiology, but every new metric also creates another opportunity for comparison, interpretation, and concern. Perfect sleep does not mean sleep without variation, yet increasingly detailed measurement can make normal variation feel like failure. Sometimes, a healthier relationship with sleep may require allowing sleep to be imperfect.
